# A 30-year bibliometric analysis of research coverage on HIV and AIDS in Lesotho

**DOI:** 10.1186/s12961-017-0183-y

**Published:** 2017-03-21

**Authors:** Eltony Mugomeri, Bisrat S. Bekele, Mamajoin Mafaesa, Charles Maibvise, Clemence Tarirai, Sunny E. Aiyuk

**Affiliations:** 1 Department of Pharmacy, Faculty of Health Sciences, National University of Lesotho, Roma Campus, P.O. Roma 180, Maseru, Lesotho; 2 Department of Nursing, Faculty of Health Sciences, University of Swaziland, Mbabane Campus, P. O. Box 369, Mbabane, Swaziland; 3 Department of Pharmaceutical Sciences, Tshwane University of Technology, Private Bag X680, Pretoria, South Africa; 4 Department of Environmental Health, Faculty of Health Sciences, National University of Lesotho, Roma Campus, P.O. Roma 180, Maseru, Lesotho

**Keywords:** Bibliometric analysis, HIV, AIDS, National research agenda, Research output

## Abstract

**Background:**

Given the well documented undesired impacts of HIV/AIDS globally, there is a need to create a statistical inventory of research output on HIV/AIDS. This need is particularly important for a country such as Lesotho, whose HIV/AIDS prevalence is one of the highest globally. Research on HIV/AIDS in sub-Saharan Africa continues to trail behind that of other regions, especially those of the developed countries. Lesotho, a sub-Saharan country, is a developing country with lower research output in this area when longitudinally compared to other countries. This study reviewed the volume and scope of the general research output on HIV/AIDS in Lesotho and assessed the coverage of the national research agenda on HIV/AIDS, making recourse to statistical principles.

**Methods:**

A bibliometric review of studies on HIV/AIDS retrieved from the SCOPUS and PubMed databases, published within the 30-year period between 1985 and 2016, was conducted. The focus of each of the studies was analysed and the studies were cross-matched with the national research agenda in accordance with bibliometric methodologies.

**Results:**

In total, 1280 studies comprising 1181 (92.3%) journal articles, 91 (7.1%) books and 8 (0.6%) conference proceedings were retrieved. By proportion, estimation of burden of infection (40.7%) had the highest research volume, while basic (5.5%) and preventive measures (24.4%) and national planning (29.4%) had the lowest. Out of the total studies retrieved, only 516 (40.3%) matched the national research agenda. Research on maternal and child health quality of care, viral load point-of-care devices, and infant point-of-care diagnosis had hardly any publications in the high priority research category of the agenda.

**Conclusion:**

Notwithstanding a considerable research output on HIV/AIDS for Lesotho, there is insufficient coverage of the national research agenda in this research area. The major research gaps on general research output are in basic and preventive measures as well as national planning. There is also a need to increase targeted funding for HIV/AIDS research to appropriately address the most compelling gaps and national needs.

## Background

Research is paramount for informing policy and intervention in any sphere of life and the importance of inventorying such cannot be overemphasised. HIV infection represents a global health concern [1]. The Joint United Nations Programme on HIV/AIDS (UNAIDS) estimates that 36.7 million people were living with HIV globally in 2015 and 1.1 million people died in the same year from AIDS resulting from HIV infection [2]. Countries in Asia and sub-Saharan Africa are the worst affected [1]. However, research on major health issues, including HIV/AIDS, in Africa and Asia continues to trail behind that of developed countries [3], mainly due to brain drain [4], lack of mentorship [5], inadequate research funding [6], and marginal collaboration [7]. To promote and strengthen research capacity in targeted research areas, including HIV/AIDS, the WHO emphasises the development and periodic review of national research agendas [8]. This is crucial, particularly for the worst affected countries, such as Lesotho.

Lesotho, located 29.6100° S, 28.2336° E, is a small landlocked country enclaved within South Africa, with a territory of about 30,000 square kilometres and a population of about 2 million [9]. The United Nations [10] notes that Lesotho is a poor country, with 40% of its population living below the official poverty line of US$1.25 per day. The country, with a 23.3% adult HIV prevalence rate, is ranked second highest in HIV prevalence worldwide [11]. HIV/AIDS have adversely affected the country’s economy and, in 2012, the Government of Lesotho spent 12% of its national budget on HIV/AIDS mitigation and treatment programmes [12].

In Lesotho, the Ministry of Health (MoH) sets the 5-year research agenda on health matters in consultation with the implementing partners, academics and civil society based on priorities of research. The current agenda (2013–2018) has 190 priority research areas classified under three major research focus areas, namely (1) health programmes and services, (2) specific health conditions and (3) support services [13]. The research focus areas are clustered into four levels of priority, defined as high, medium, low and not urgent. HIV/AIDS, classified under the specific health conditions research focus area, is one of the key high-level priority areas for the MoH. Due to the problem of HIV/AIDS in Lesotho, the 2013–2018 national research agenda allocated a conspicuous place to HIV/AIDS research [13]. Thus, by proportion, research areas on HIV/AIDS dominate the agenda, constituting 16% of the 190 priority research areas. However, the volume and scope of the general research output on HIV/AIDS, and the coverage of the set research goals in the country remain, at best, undescribed. This lack of evidence on the progress of research on HIV/AIDS affects the national research focus and evidence-based policymaking. As 2018 approaches, the year when the present health research agenda term for the country ends, a bibliometric review of the literature on HIV/AIDS in Lesotho seems imperative.

The purpose of this study was to assess the volume and scope of the general research output on HIV/AIDS in Lesotho and to evaluate the coverage of the national research agenda on HIV/AIDS. The overarching aim was to highlight research areas on HIV/AIDS that are on this agenda which need to be strengthened.

## Methods

### Study design

**Fig. 1:**
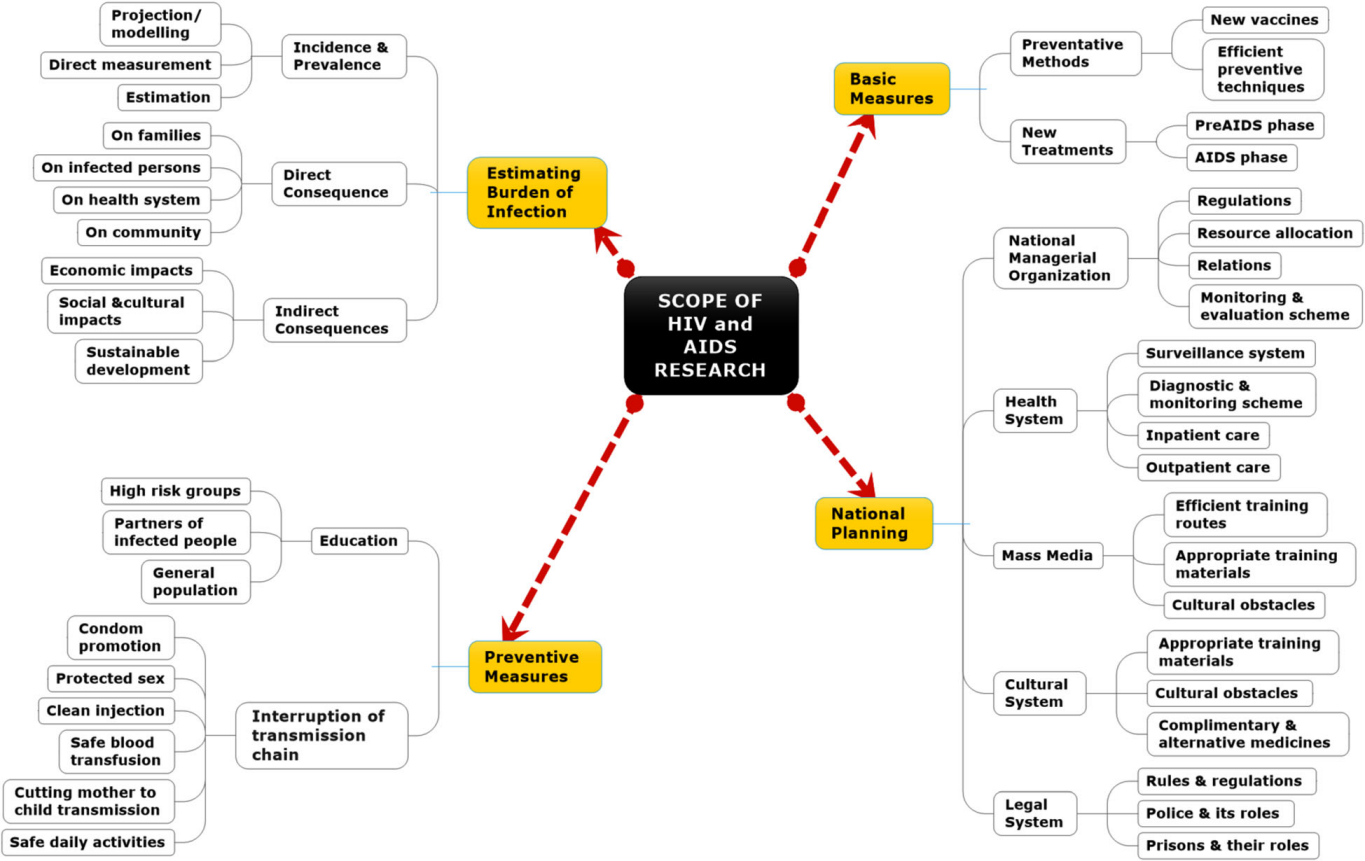
Conceptual framework for classifying the scope of research on HIV/AIDS. The four major categories of the scope are classified into sub-categories and each sub-category is further classified into elements. Source: Adapted from Haghdoost et al. [1]

A bibliometric search of research output on HIV/AIDS from Lesotho, published between 1985 and 2016, covering a 30-year period, was conducted to establish the volume and scope of the publications, and the extent of coverage of the national research focus on HIV/AIDS. Bibliometric studies systematically evaluate research output and analyse temporal and geographical distribution of research output on a specific topic [14] and lend such output to statistical analyses. A conceptual framework for benchmarking the general research output and assessing research progress in a specific area is important. This study used a conceptual framework, as outlined by Haghdoost et al. [1], to analyse and classify the scope of research on HIV/AIDS. The framework classifies HIV/AIDS research into four key priority areas, namely prevention, planning, epidemiology (burden estimation) and basic research or measures (Fig. 1).

### Data abstraction and analysis

Published scientific literature that met the inclusion criteria were retrieved from SCOPUS and PubMed database and made into a bibliographic list in EndNote® version X7.2. The inclusion criteria for the retrieval were based on search terms derived from elements in the adapted conceptual framework for evaluating the general scope of research on HIV/AIDS (Fig. 1). Each element in the conceptual framework was translated into a search term (Table 1). The search terms were then used in SCOPUS and PubMed. However, for searching in PubMed, the search terms were transformed into medical subject headings (MeSH), where appropriate. For example, the search term, “New preventive methods AND vaccines” used in PubMed was “New preventive methods” AND “vaccines”[MeSH]”. The SCOPUS and PubMed databases provide a wide coverage of journals with considerable quality [15], while EndNote is a referencing software which is used to generate bibliographic lists in works that include research papers, theses and dissertations [16]. MeSH terms are precise vocabulary used for indexing journal articles and books in the life sciences to facilitate search in PubMed [17].

**Table 1:** List of primary search terms used to retrieve suitable studies

Basic measures^a^	Estimating burden of infection^a^	National and subnational planning^a^	Preventative measures^a^
New preventive methods AND^b^ vaccines^c^	Burden of infection on community	Education system AND cultural obstacles	Education AND general population
New preventive methods OR^b^ techniques	Burden of infection on families	Cultural system AND HIV complementary and alternative medicine	Education AND HIV high risk groups
New treatment OR Drugs AND AIDS phase	Burden of infection on health system	Education system AND training materials	Education AND partners of infected people
New treatment OR Drugs AND Pre-AIDS phase	Burden of infection on infected cases	Health system AND diagnostic and monitoring scheme	Transmission AND clean injection
	Incidence OR prevalence AND direct measurement	Health system AND outpatient cares	Transmission AND condom promotion
	Incidence OR prevalence AND estimation	Health system AND inpatient cares	Cutting mother to child transmission
	Incidence OR prevalence projection or modelling	Health system OR surveillance system	Transmission AND protected sexual contact
	Burden of infection AND economic impacts	Legal system AND police and its roles	Transmission AND safe blood transfusion
	Burden of infection AND social and cultural impacts	Legal system AND prisons and their roles	Transmission AND safe daily activities
	Burden of infection AND sustainable development	Legal system AND rules and regulations	
		Mass media AND the most efficient training routes	
		Mass media AND the most appropriate training materials	
		Mass media AND the most important cultural obstacles	
		Managerial AND regulations	
		Managerial AND relations	
		Managerial AND resource allocation	
		Managerial AND monitoring and evaluation scheme	

The above search terms were used in SCOPUS and PubMed. All search terms also included the term "AND Lesotho" For searching in PubMed and where appropriate, the search terms were transformed into medical subject headings (MeSH). For example, the search term, “New preventive methods AND vaccines” used in PubMed was “New preventive methods” AND “vaccines”[MeSH]”

^a^Categories for the scope of research on HIV/AIDS

^b^Boolean logic term for searching databases

^c^Primary search terms were derived from text words of each element in the conceptual framework

**Fig. 2:**
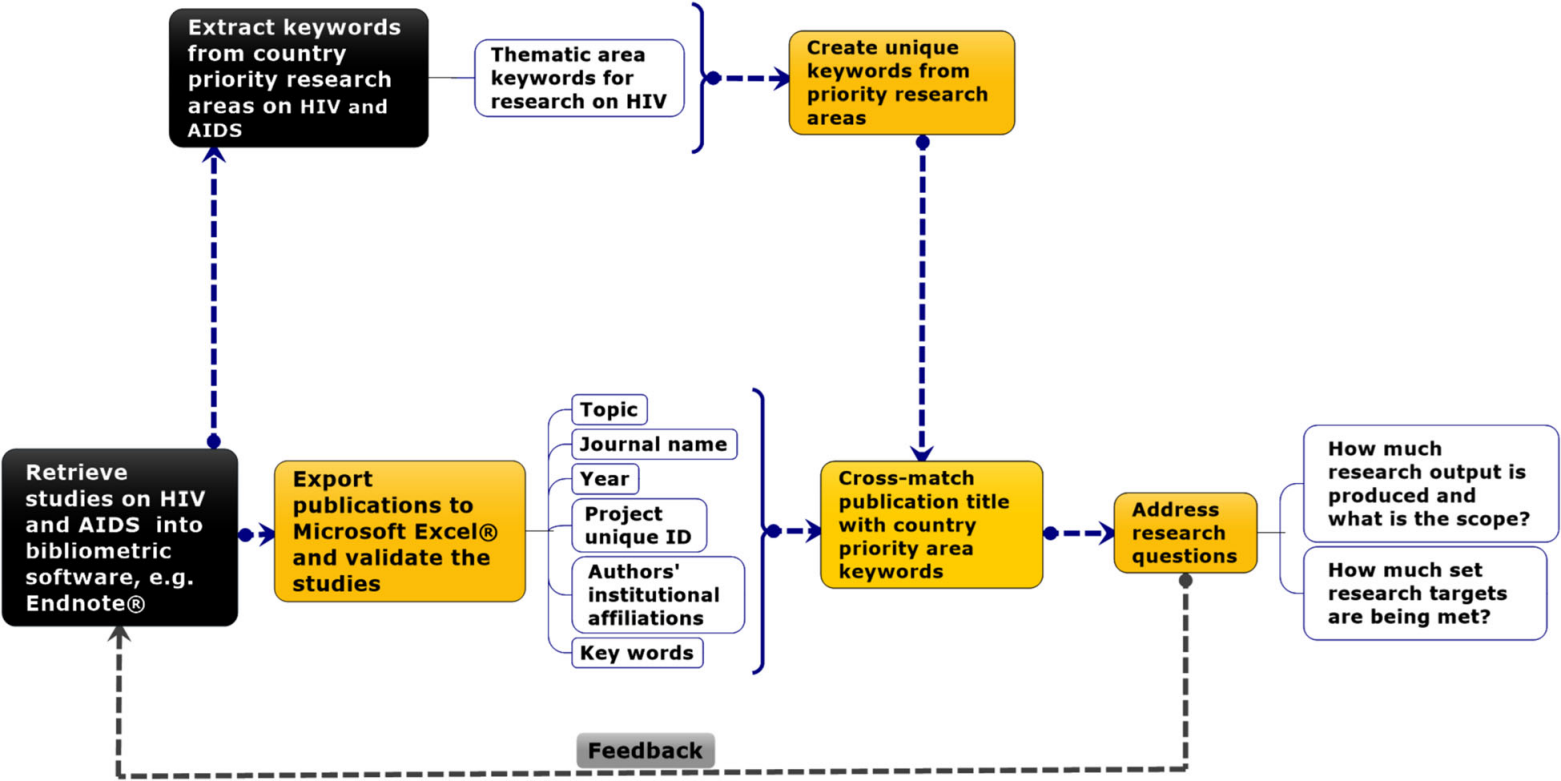
The study layout for evaluating the volume and scope of research on HIV/AIDS

The retrieved study topics, their abstracts and the associated meta-data were exported from EndNote software to Microsoft Excel® for manual validation and classification. Each validated study was classified into one single most suitable element of the adapted scope of research on HIV/AIDS. The retrieved studies were then cross-matched to the priority area keywords derived from the national research agenda according to the criteria presented in Fig. 2. The cross matching was performed using Matchit*®* in Stata version 13 and validated using manual discretion in Microsoft Excel® version 15. Matchit*®* is a text matching software tool that gives a matching score based on a pre-determined threshold in Stata [18].

## Results

### Volume and trend of research output on HIV/AIDS (1985–2016)

**Fig. 3:**
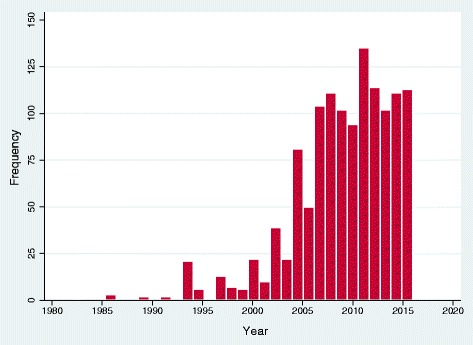
Research volume on HIV/AIDS in Lesotho from 1985 to 2016 (n = 1280)

In total, 1280 scientific works comprising 1181 (92.3%) journal articles, 91 (7.1%) books and 8 (0.6%) conference proceedings were retrieved. Figure 3 presents the annual number of retrieved studies published between 1985 and 2016. Research volume was low between 1985 and 2003, but reached a peak in 2011. The research volume remained fairly constant after 2011 (Fig. 3).

### Volume and scope of research on HIV/AIDS in Lesotho (1985–2016)

**Fig. 4:**
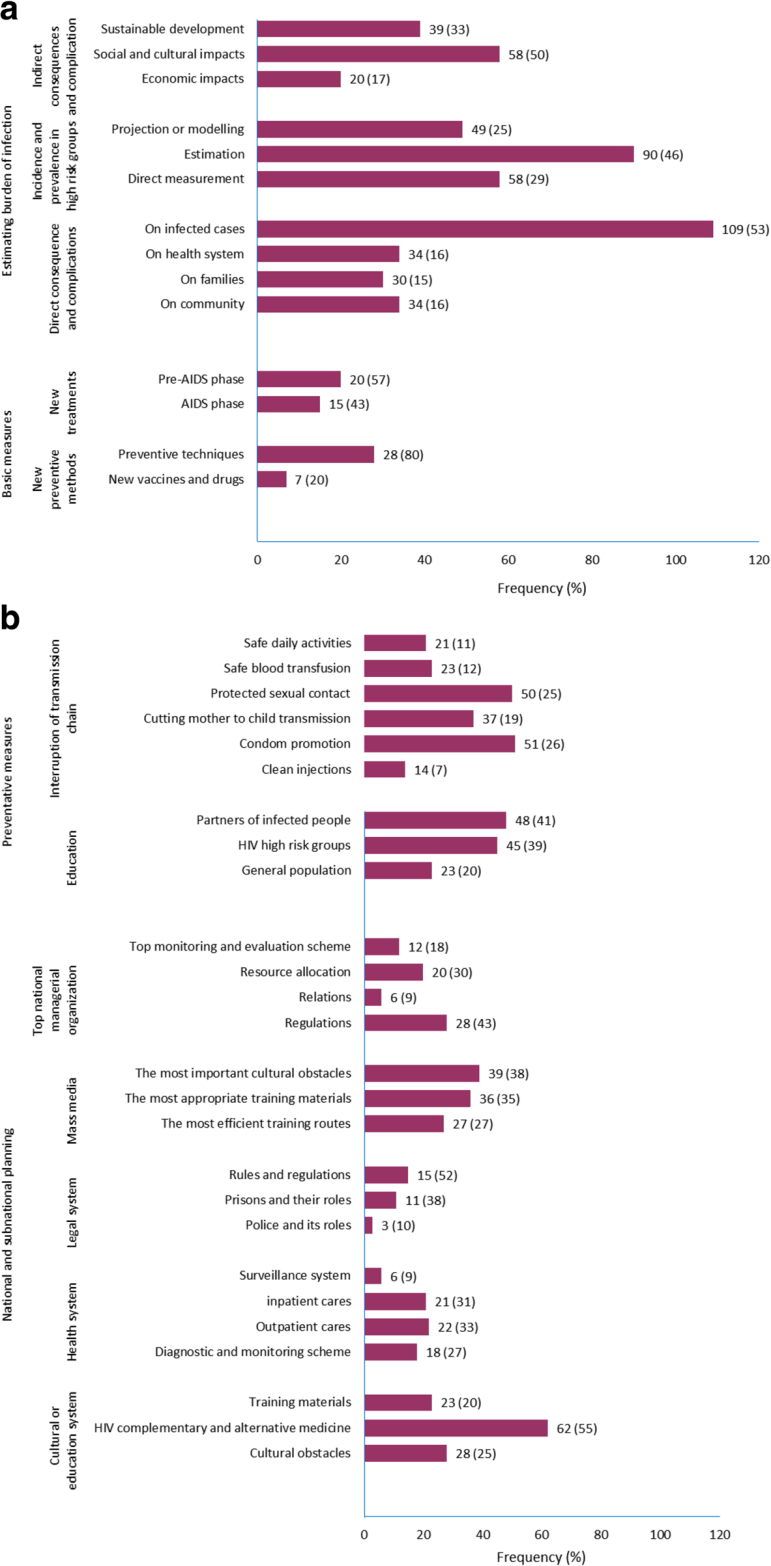
**a** Frequency and percentage (%) of retrieved studies in the categories of basic measures and estimating the burden of infection. The denominator used to calculate the percentages was n = 1280 studies. Bars represent frequency of studies retrieved per category. Figures on the bar graphs indicate frequency and percentage (%). **b** Frequency and percentage (%) of retrieved studies in the categories of national planning and preventative measures. The denominator used to calculate the percentages was n = 1280 studies. Bars represent frequency of studies retrieved per category. Figures on the bar graphs indicate frequency and percentage (%)

Figure 4a and b jointly present the volume and scope of research on HIV/AIDS in Lesotho, over the study period reviewed. By proportion, basic (5.5%) and preventive measures (24.4%), and national planning (29.4%) had the lowest volume. However, estimation of the burden of infection (40.7%) had the highest volume.

Within the category of basic measures, research on new vaccines and drugs, with only seven publications [19–25], had the lowest research volume (Fig. 4a), while research on methodologies of interrupting HIV transmission, particularly the use of clean injections and other invasive devices, had only 14 publications in the preventive measures category (Fig. 4b). In the national planning category, research on the police force and its role in preventing the spread of HIV had the least research volume, with only three publications [26–28]. In addition, research on surveillance [29–34] and collaborative relations [35–40] between different national task forces on HIV/AIDS, with only six publications each, had the least number of publications in the national planning category.

### Research coverage of national priority areas on HIV/AIDS in Lesotho

**Fig. 5:**
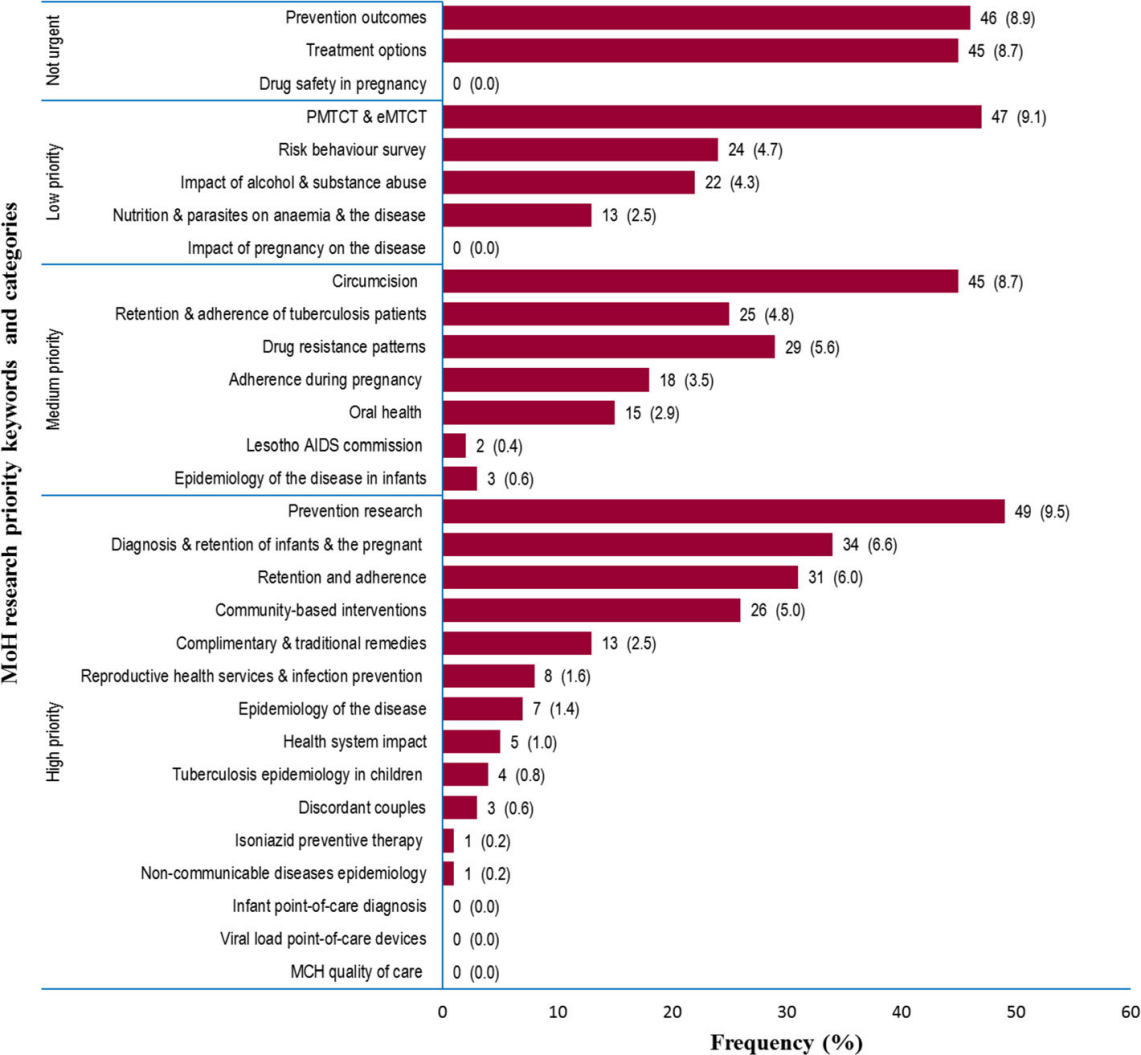
Frequency and percentage (%) of studies addressing the 2013–2018 national research agenda on HIV/AIDS in Lesotho (n = 516). Bars represent frequency of studies retrieved per category. Figures on the bar graphs indicate frequency and percentage (%). *MCH* mother and child health; *MoH* Ministry of Health; *PMTCT* prevention of mother-to-child transmission; *eMTCT* elimination of mother-to-child transmission

Out of the 1280 studies retrieved, only 516 (40.3%) matched the national research agenda. Figure 5 presents the research coverage of the national research agenda on HIV/AIDS in Lesotho. Fourteen research priority areas in the national agenda had less than 10 publications. Among these, research on maternal and child health quality of care, viral load point-of-care devices, and infant point-of-care diagnoses had hardly any publications in the high-priority research category. In addition, research on epidemiology of non-communicable diseases in people living with HIV [41], uptake and effectiveness of isoniazid preventive therapy for opportunistic tuberculosis infection [42], and discordant transmission of HIV in couples [20, 43, 44] had the least number of publications in the same category. Moreover, research on epidemiology of tuberculosis in children [45–48] and impact of HIV on the national health system [20, 49–52] had a low number of publications in the high-priority research category. However, research on the impact of pregnancy on HIV progression and drug safety during pregnancy had hardly any publications in the low- and non-urgent-priority areas.

## Discussion

The fact that out of the 1280 total publications retrieved, research on basic measures, particularly on new vaccines and drugs, had the lowest number of publications emphasises the need to strengthen basic research in Lesotho. However, Scott et al. [53] note that lack of funding for basic research is a major obstacle in developing countries, although Lesotho has a fairly high research volume with a very uneven spread. Over-strained public budgets in developing countries rarely support basic research. Scott et al. [53] further argue that, globally, basic research is predominantly supported by public funds while the private sector mainly funds applied research and technology development, clearly for its own benefit. Similarly, the research gaps on preventive measures, including on the methodologies of interrupting HIV transmission, identified in this study highlight the need to increase focus on this research area. Moreover, the need to increase efforts on research in national planning and its sub-elements cannot be overemphasised.

The fact that only 40.3% of the retrieved studies matched the national research agenda implies that a considerable volume of research outputs on HIV/AIDS in Lesotho does not address the national research agenda in this research area. This emphasises the need to strengthen the national research focus in this study area. One way of achieving this goal may be by conducting workshops with researchers to sensitise them about the priority research areas. Research areas with hardly any publications identified in this study need urgent attention, with the possible need of special funding and incentives to bridge the gaps. Literature on long-term strategies to stimulate research in targeted areas is abundant. The strategies include increasing targeted research funding to researchers [6], tackling mentorship challenges through retaining experienced researchers [5], and encouraging research collaborations [7].

This study was focused on and limited to the bibliometric analysis of the research output on HIV/AIDS. The study did not assess the programmatic impact of the research studies included in this review, which could be a potentially interesting future study. It is important to note that this study investigated the research output on HIV/AIDS in Lesotho based on absolute numbers of publications retrieved using pre-defined search criteria. Therefore, the quality of studies classified herein as addressing particular research priority areas cannot be entirely guaranteed, neither can the overall comprehensiveness of the literature used. However, this limitation is intrinsic to all bibliometric studies [54, 55]. Furthermore, the conceptual framework adopted for analysing and classifying the scope of research on HIV/AIDS in this study is not exhaustive, while the scope of research led by civil society and people living with HIV in Lesotho is limited mostly to secondary participation; as such, these research areas were not dealt with in this paper. Consequently, studies promoting the application of broader frameworks for classifying the scope of research on HIV/AIDS are recommended.

Comparative analysis of research outputs within and between different countries needs caution. McKee et al. [3] note that such analysis is also affected by a lack of comparable international indicators for research outputs. The authors further argue that the gross national product per capita and total health expenditure should be factored in the comparative analysis of research output across countries. Therefore, this study should be interpreted in the context of Lesotho and may only be extrapolated to other countries with comparable international indicators of research outputs.

As the year 2018 approaches and the set period for the national research agenda comes to an end, this study is useful, particularly for the MoH, in reviewing the research progress made on HIV/AIDS, setting priority research areas for the subsequent national research agendas and for national and sub-national allocation of research funds. This study may also stimulate and channel academic research towards the national research agenda of Lesotho. In addition, implementing partners working on HIV/AIDS projects in the country may find this study useful in identifying research areas that need to be prioritised for funding. Furthermore, this study augments the efforts by the WHO on its drive to encourage the development and periodic review of national research agenda on HIV/AIDS in regions worst affected by the disease, particularly in sub-Saharan African countries [8].

## Conclusion

The study revealed that there is a considerable research output on HIV/AIDS in Lesotho. However, there is insufficient coverage of the national research agenda on HIV/AIDS. The major research gaps are in basic and preventive measures as well as national planning. Some research areas in the national research agenda on HIV/AIDS are hardly covered by a single study, including high priority research areas. The current study therefore highlights the need to increase targeted funding for HIV/AIDS research, especially towards the research areas with the most compelling gaps and national needs. The present study also delineates focus areas for research within its theme.

## Abbreviations

MeSH: Medical Subject Headings
MoH: Ministry of Health
